# Fault Diagnosis of a Rolling Bearing Based on Adaptive Sparest Narrow-Band Decomposition and RefinedComposite Multiscale Dispersion Entropy

**DOI:** 10.3390/e22040375

**Published:** 2020-03-25

**Authors:** Songrong Luo, Wenxian Yang, Youxin Luo

**Affiliations:** 1 Hunan Provincial Cooperative Innovation Center for the Construction & Development of Dongting Lake Ecological Economic Zone, Changde 415000, China; llyx123@126.com; 2 College of Mechanical Engineering, Hunan University of Arts and Science, Changde 415000, China; 3 School of Engineering, Newcastle University NE1 7RU, UK; wenxian.yang@ncl.ac.uk

**Keywords:** adaptive sparest narrow-band decomposition, multiscale analysis, refined composite multiscale dispersion entropy, fault diagnosis

## Abstract

Condition monitoring and fault diagnosis of a rolling bearing is crucial to ensure the reliability and safety of a mechanical system. When local faults happen in a rolling bearing, the complexity of intrinsic oscillations of the vibration signals will change. Refined composite multiscale dispersion entropy (RCMDE) can quantify the complexity of time series quickly and effectively. To measure the complexity of intrinsic oscillations at different time scales, adaptive sparest narrow-band decomposition (ASNBD), as an improved adaptive sparest time frequency analysis (ASTFA), is introduced in this paper. Integrated, the ASNBD and RCMDE, a novel-fault diagnosis-model is proposed for a rolling bearing. Firstly, a vibration signal collected is decomposed into a number of intrinsic narrow-band components (INBCs) by the ASNBD to present the intrinsic modes of a vibration signal, and several relevant INBCs are prepared for feature extraction. Secondly, the RCMDE values are calculated as nonlinear measures to reveal the hidden fault-sensitive information. Thirdly, a basic Multi-Class Support Vector Machine (multiSVM) serves as a classifier to automatically identify the fault type and fault location. Finally, experimental analysis and comparison are made to verify the effectiveness and superiority of the proposed model. The results show that the RCMDE value lead to a larger difference between various states and the proposed model can achieve reliable and accurate fault diagnosis for a rolling bearing.

## 1. Introduction

The reliability of a rolling bearing plays a vital role in ensuring stable and reliable operation of a mechanical system. If local failure of a rolling bearing is not detected as early as possible, it is likely to cause a breakdown of a mechanical system or major production safety accidents, resulting in huge economic losses. Therefore, condition monitoring and fault diagnosis fora rolling bearing have become a prevalent topic in this scientific research field [1,2,3,4,5,6,7]. 

Due to the influence of nonlinear factors such as varying load, clearance, nonlinear stiffness, friction, vibration signals of a rolling bearing present nonlinear and nonstationary characteristics. Therefore, it is essential to adopt an adaptive signal analysis method to extract hidden patterns or physical information. At present, various advanced signal processing techniques including wavelet transform [2] (WT), empirical mode decomposition (EMD), and its improved version [8,9,10,11], local mean decomposition (LMD) [3,12], variation mode decomposition (VMD) [13,14], matching pursuit (MP) [15,16], have been used to vibration signals analysis. WT and VMD are the most prevalent techniques, but WT needs to have a pre-determined wavelet basis that will have a great influence on the results. The effectiveness of the VMD algorithm depends on its parameters to some extent, which may reduce the analysis capability of VMD. Empirical mode decomposition can adaptively decompose a nonstationary signal into a series of intrinsic mode functions (IMFs). However, in EMD and its improvements: ensemble empirical mode decomposition (EEMD) and complementary ensemble empirical mode decomposition (CEEMD) [9], there are still some problems needing to be addressed, such as mode mixing, and end-point effect. Matching pursuit (MP) based on compressive sensing theoryis an excellent signal process method, which uses highly redundant dictionary to obtain the sparsest representation of signals. Nevertheless, the decomposition results are usually lacking in physical meaning. Inspired by EMD and compressive sensing theory, Hout Y and Shi ZQ proposed an adaptive sparest time-frequency analysis (ASTFA)method in [17]. The main principle of the ASTFA method is to search for the sparest components from a highly redundant dictionary library that includes intrinsic mode functions (IMFs). Each component obtained by ASTFA is the product of an envelope function and a cosine function. The constraint is that the envelope function is smoother than the cosine function, so that the instantaneous frequency of each component has explicit physical meaning. The ASTFA uses an optimization technique to derive the intrinsic components instead of fitting the envelope of extreme points and their sifting progress in the EMD. Therefore, the ASTFA can tackle limitations in EMD. Nevertheless, the ASTFA algorithm requires massive computational costs because of the optimization process. The Gauss–Newton optimization technique is a fast technique that was used in the literature [17]. However, the Gauss–Newton algorithm is sensitive to initial values, leading to inaccurate results. To address these problems, Cheng proposed an improved ASTFA method, named adaptive sparsest narrow-band decomposition method (ASNBD) [18]. A signal can be decomposed into several intrinsic narrow-band components (INBCs). In addition, the simulation results show that ASNBD not only inherits the advantages of ASTFA but also improves the decomposition accuracy and stability.

On the other hand, when local failure exists in a rolling bearing, the complexity of the intrinsic oscillation modes hidden in the vibration signals will differ from that under normal state. Many nonlinear dynamic parameter estimations have been utilized as feature extraction approaches, among which, correlation dimension, entropy-based measures are the most popular techniques. However, the reliable estimation of correlation dimension requires long-term time series, which brings great limitation when short-term vibration signals are analyzed. Entropy-based measures include sample entropy (SampEn), fuzzy entropy (FE), permutation entropy (PE), and so on. However, initial entropy-based measures only complete single-scale analysis, which generally assign the highest values to highly unpredictable random signals but not structurally complex signals. Hence, single-scale entropy measures cannot physically quantify the complexity of time series [19].The multiscale entropy (MSE) algorithm was proposed by Costa in [19,20] and applied to rolling bearing fault diagnosis firstly in [21]. However, traditional multi-scale entropy algorithm would shorten the dataset and yield undefined values for short-term data when big scale factors are adopted. To alleviate these deficiency, Wu et al. proposed modified multiscale entropy [22], which employed a moving-average algorithm to acquire more template vectors. However, this modified multiscale algorithm vastly increases computation time. Later, composite multiscale sample entropy (CMSE) [23] and refined composite multiscale sample entropy (RCMSE) [24] were developed for a new coarse-graining procedure, in which different start points were utilized. As an improved version of sample entropy, fuzzy entropy was proved more robust to noise and less sensitive to data length and parameters. Inspired by literature [23,24], composite multiscale fuzzy entropy were introduced to extract the nonlinear features of vibration signals of a rolling bearing [7]. Recently, Hamed Azami proposed refined composite multiscale fuzzy entropy (RCMFE)based on standard deviation and successfully applied to biomedical signal analysis [25]. However, both RCMSE and RCMFE may still produce undefined values for short-term datasets. PE and multiscale permutation entropy (MPE) are based on the permutation patterns or the order relations of the amplitude of a signal, and have been used to fulfill machinery fault diagnosis tasks [2,3,26,27]. Nevertheless, the mean of amplitudes and differences between amplitude values is not considered [28,29]. Recently, refined composite multiscale dispersion entropy (RCMDE) has been developed and the application results to synthetic datasets and real-world biomedical signals show that the RCMDE is more stable than the RCMSE algorithm in [29].

By making use of the advantages of the ASNBD and the RCMDE, a novel-fault diagnosis-model for a rolling bearing is proposed in this paper. Firstly, the vibration signal collected is decomposed into a number of INBCs using ASNBD technique. Following that, several relevant INBCs including rich fault information are used to extract RCMDE values as features. Lastly, basic Multi-Class Support Vector Machine (multiSVM) is employed to fulfill the fault identification. Simultaneously, the effectiveness and superiority of the proposed method is verified by experimental datasets. The remaining part is organized as follows. In Section 2, the adaptive sparsest narrow-band decomposition method is introduced. In Section 3, the algorithm of refined composite multiscale dispersion entropy is described. A novel-fault diagnosis-model is presented in Section 4, and applied in Section 5. The conclusions are given in Section 6. 

## 2. Adaptive Sparsest Narrow-Band Decomposition Method

### 2.1. ASTFA Brief

Since the ASTFA method is the basis of ASNBD, the algorithm of ASTFA is summarized as shown below:

**Step 1:** Construct a highly redundant dictionary Dic:

(1) $$Dic=\{a(n)\cos(\theta (n)):\theta^{\prime }(n)\geq 0,\;a(n)\in V(\theta )\}$$

$$V(\theta )=Span\{\cos(\frac{k\theta }{n}),\sin(\frac{l\theta }{n}):k=0,\cdots \lambda n,l=1,\cdots \lambda n\}$$

where, $\theta^{\prime }(n)\geq 0$ is to guarantee instantaneous frequencies of IMFs being physical meaning. a(n)∈V(θ) can ensure that the envelop function a(n) is smoother than the cosine function.

**Step 2:** Search for the sparest decomposition of the original signal x(n) by iterative operation in optimization process as follows:

(1) Set $i=1,r_{0}(n)=x(n)$

(2) Solve the optimization problem P1 with nonlinear constraint using Gauss–Newton algorithm.

(2) $$P1:\;Minimize{\left\|r_{i}(n)-IMF_{i}(n)\right\|}_{2}^{2}$$

$$\mathrm{Subject}\;\mathrm{to}\;IMF_{i}(n)\in Dic$$

(3) Set $r_{i}(n)=r_{i-1}(n)-IMF_{i}(n)$

(4) If ${\left\|r_{i}(n)\right\|}_{2}^{2}\leq \varepsilon$ is satisfied, stop the program and obtain the decomposition results; otherwise, let i=i+1 and return to sub-step (2) to repeat until termination condition is met.

From the above process, it can be seen that the ASTFA does not depend on the distribution of extreme points. Hence, it can inhibit some deficiency caused by the fitting processing of extreme points in EMD. Moreover, the ASTFA algorithm has a solid mathematical foundation. In the literature, the Gauss–Newton algorithm was adopted to solve the optimization problem P1 to search for the sparest represent components in the literature [17]. However, Gauss–Newton algorithm highly depends on the initial values. If the initial values deviate too far from the real values, the solution often diverges after iteration and the inaccurate components may appear.

### 2.2. ASNBD Algorithm

To overcome the shortcomings of ASTFA method, the ASNBD algorithm is introduced to complete nonstationary signal process in this paper. In the ASNBD, a filter with optimization parameters is built by solving a nonlinear optimization problem, and a regulated differential operator is used as the objective function so that each component is constrained to be a local narrow-band signal to generate an intrinsic narrow-band component (INBC). Furthermore, the immune genetic algorithm [30] (IGA) is utilized to address a nonlinear optimization instead of Gauss–Newton algorithm. In order to depict the ASNBD method, the definition of intrinsic narrow-band signal is illustrated firstly as follow. 

For a signal expressed as A(t)cos(ωt+ϕ(t)), if its phase function ϕ(t) varies slowly and its amplitude function A(t) is band-limited, and the maximal frequency of A(t) is much smaller than ω, it can be defined as a narrow-band signal. Furthermore, if a neighborhood interval exists at any point of the signal, the signal can be regards as a local narrow-band signal. A singular local linear operator will converted a local narrow-band signal to zero [31]. In this paper, a singular local linear operator T, developed in the literature [31], will be adopted as shown below:

(3) $$T={\left(\frac{1}{\omega^{2}}\frac{d^{2}}{dt^{2}}+1\right)}^{2}$$

Similar to the ASTFA algorithm, after constructing a highly redundant dictionary Dic as Equation (1), the ASNBD algorithm will search for the sparest INBCs by solving the optimization problem P2 with nonlinear constraint. The ASNBD algorithm is illustrated as shown below [18]:

(1) Set $i=1,r_{0}(n)=x(n)$;

(2) Solve the following nonlinear constrained optimization problem P2:

(4) $$\mathrm{Minimize}\;{\left\|T(INBC_{i}(n))\right\|}_{2}^{2}+\lambda {\left\|D(r_{i}(n)-INBC_{i}(n))\right\|}^{2}$$

$$\mathrm{Subject}\;\mathrm{to}\;x(n)=\sum_{i=1}^{M}INBC_{i}(n)+residue,i=1,\ldots ,M$$

where M is the number of INBCs; T is the differential operator as Equation (3); D is an operator that regulates the residue; λ is the weight of ${\left\|T(INBC_{i}(n))\right\|}_{2}^{2}$ and ${\left\|D(r_{i}(n)-INBC_{i}(n))\right\|}^{2}$, and, in general, λ is set to 1. 

(3) Set $r_{i+1}(n)=r_{i}(n)-INBC_{i}(n)$

(4) If ${\left\|r_{i+1}(n)\right\|}_{2}^{2}\leq \varepsilon$, then stop; otherwise, set i=i+1 and go to the step (2).

The optimization objective function is that INBC(n) is constrained to be a local narrow-band signal. Thus, the obtained INBCs have explicit physical meaning in ASNBD. However, the optimization of all data points requires a massive computational cost, especially when the dataset size is big. In order to reduce the computational tension, in step (2), the optimization of all data points can be transformed into the optimization of the parameter vector β of a filter χ [18]. In other words, the sparest INBCs can be obtained by solving the optimization calculation $P_{3}$ for the parameter vector β of a filter χ. IGA is an improved genetic algorithm, which can effectively improve population diversity and restrain premature convergence of traditional genetic algorithm due to the combination of biological immune mechanism and genetic algorithm. On the one hand, in the immune system, antibodies promote or inhibit each other to maintain population diversity. On the other hand, large-scale optimization calculation is carried out through immune selection, immune variation, immune update, and new dynamic adjustment operation. Moreover, IGA adopts immune memory function, which improves the overall search ability and speeds up the search procedure. In addition, IGA is not sensitive to the initial values. Accordingly, IGA is used to address the optimization problem $P_{3}$ (as shown below) for the parameter vector β of a filter χ. The procedure of the optimization calculation is depicted as shown below.

(1) Calculate the fast Fourier transformation ${\hat{r}}_{i}(k)$ of $r_{i}(n)$.

(2) Design a filter $\chi (k|\beta )\;(\beta =[\omega ,\omega_{b},\omega_{c}])$:

(5) $$\chi (k|\lambda )=\left\{ \begin{array}{ll} \sin\omega \left[k-\omega_{c}+\omega_{b}+\pi /(2\omega )\right], & \omega_{c}-\omega_{b}-\pi /(2\omega )\leq k<\omega_{c}-\omega_{b}\\ 1, & \omega_{c}-\omega_{b}\leq k\leq \omega_{c}+\omega_{b}\\ \cos\omega (k-\omega_{c}-\omega_{b}), & \omega_{c}+\omega_{b}<k\leq \omega_{c}+\omega_{b}+\pi /(2\omega )\\ 0, & \mathrm{else} \end{array}\right.$$

**Figure 1:**
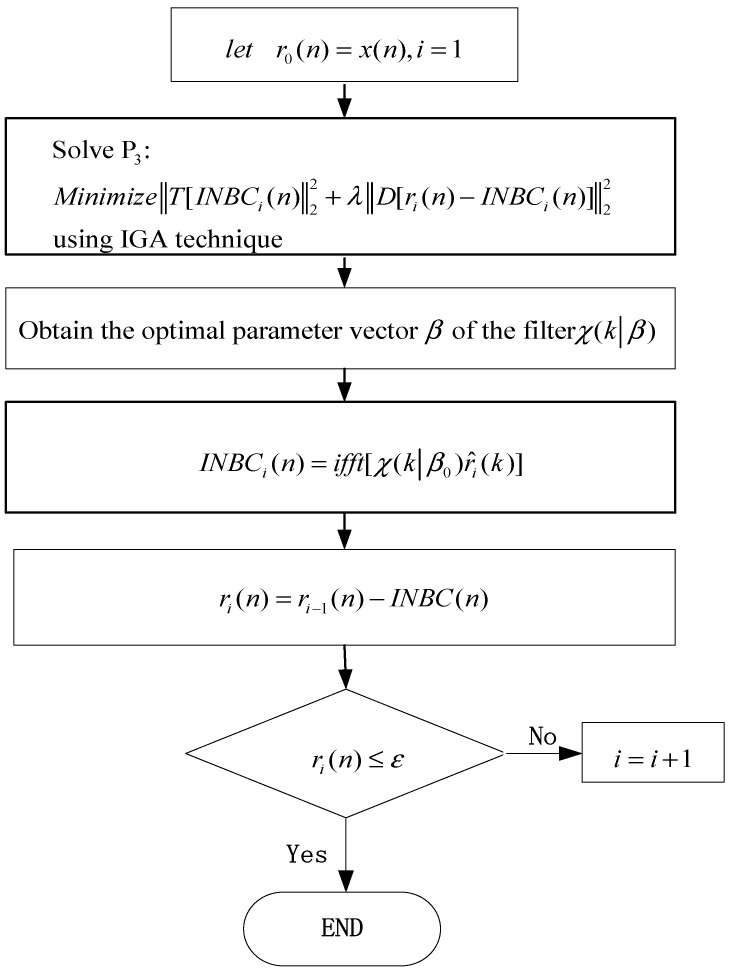
Flowchart of the adaptive sparest narrow-band decomposition (ASNBD) algorithm.

(3) Solve the following nonlinear unconstrained optimization problem $P_{3}$ to obtain parameter vector $\beta_{0}$ of a filter χ(k|β) by applying IGA algorithm. The initial values are created randomly in the IGA algorithm, and the maximum number of generations is set to 200, and the termination tolerance is e–6 and the population size is 500 in the IGA procedure. The flowchart of the ASNBD algorithm is given in Figure 1.

(6) $$P_{3}:\;\mathrm{Minimize}\;{\left\|T\left\{ifft\left[\chi (k|\beta ){\hat{r}}_{i}(k)\right]\right\}\right\|}_{2}^{2}+\lambda {\left\|D(r_{i}(t)-ifft\left[\chi (k|\beta ){\hat{r}}_{i}(k)\right])\right\|}^{2}$$

(4) Convert the filter with optimized parameter vector $\beta_{0}$ to $INBC_{i}(n)$ using inverse fast Fourier transformation. In fact, the $INBC_{i}(n)$ is obtained through the filtering process using the optimal filter designed in step (3).

(7) $$INBC_{i}(n)=ifft\left[\chi (k|\beta_{0}){\hat{r}}_{i}(k)\right]$$

### 2.3. Simulation Analysis for ASNBD

**Figure 2:**
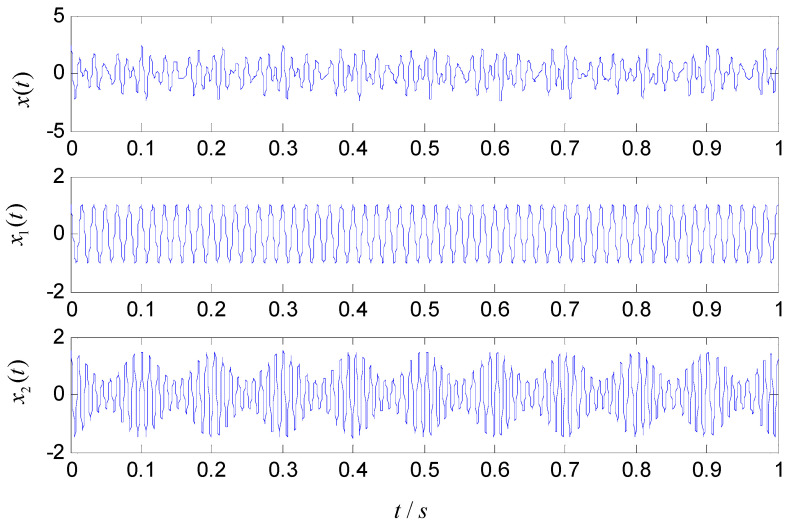
Time-domain waveforms of simulation signal.

A simulation signal x(t) is used to verify the effectiveness and superiority of the ASNBD technique. x(t) includes a cosine signal $x_{1}(t)$ and an amplitude-modulated and frequency- modulated (AM–FM) signal $x_{2}(t)$. The time domain waveforms of x(t) and its components are shown in Figure 2 and is written as shown below.

(8) $$\{\begin{array}{c} x(t)=x_{1}(t)+x_{2}(t)\\ x_{1}(t)=\cos(120\pi )\\ x_{2}(t)=[1+0.5\sin(20\pi t)]\sin(180\pi t+\cos(20\pi t^{2})) \end{array}$$

**Figure 3:**
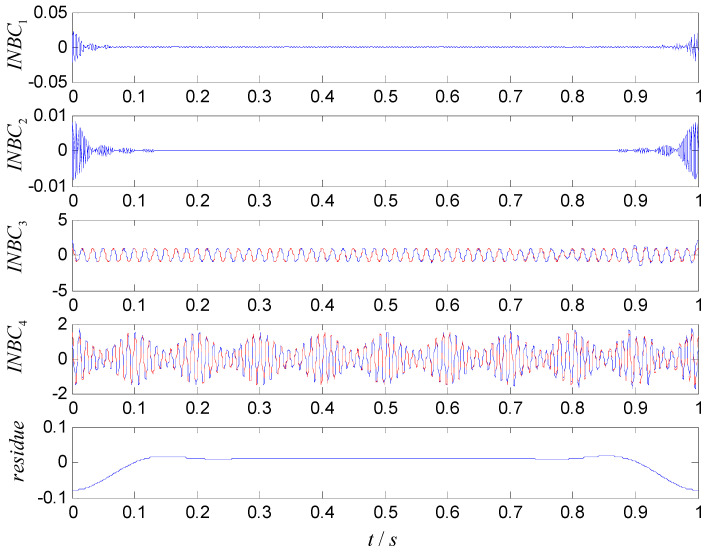
Obtained results using the ASNBD method (the blue lines indicate the decomposed results and the red lines show the true components).

**Figure 4:**
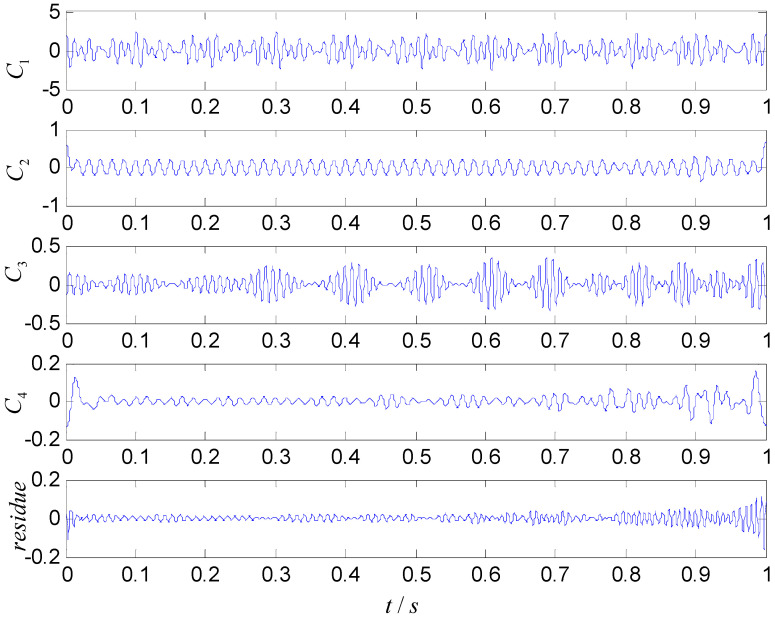
Obtained results using the adaptive sparest time frequency analysis (ASTFA) method.

**Figure 5:**
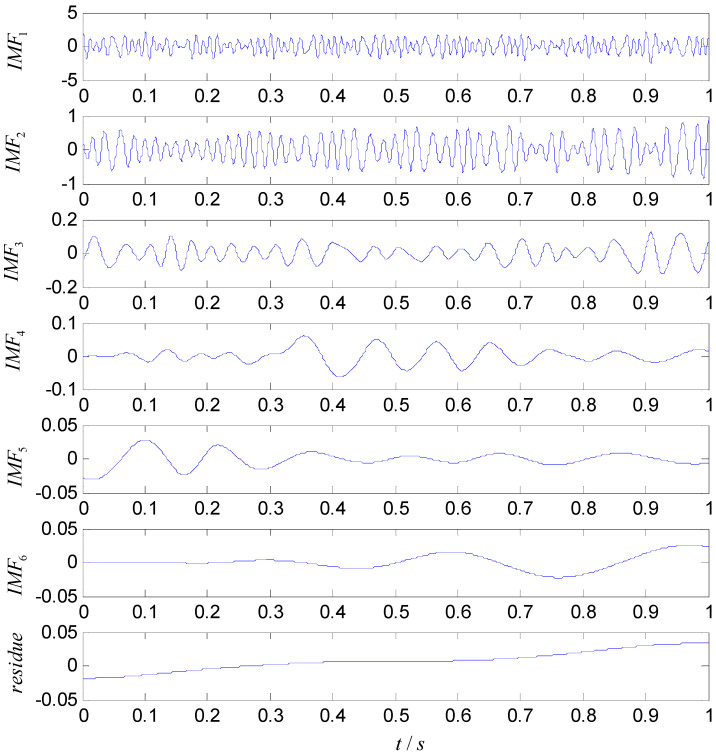
Obtained results using the complementary ensemble empirical mode decomposition (CEEMD) method.

For comparison, ASNBD, ASTFA, and CEEMD are utilized to analyze the signal x(t). The results are shown in Figure 3, Figure 4 and Figure 5, respectively. In Figure 3, the first two components are obviously false high-frequency components with weak energy only at the two end-point part, which may be generated due to the decomposition procedure, and the third component $INBC_{3}$ and the fourth component $INBC_{4}$ are consistent with the true components. Therefore, the two components are very useful component. Although the obtained components using ASTFA technique also reflect the real components (shown as $C_{2}$,$C_{3}$) in Figure 4, their energies reduce a lot and their waveforms exhibit a big deviation relative to the true component. From Figure 5, it can be seen that the real component sare not successfully derived by the CEEMD technique. At the same time, the further calculation shows that the correlation coefficient of $INBC_{3}$ and $x_{1}(t)$ is 0.9824, and correlation coefficient of $INBC_{4}$ and $x_{2}(t)$ is 0.9875 by using ASNBD, while correlation coefficient of $C_{2}$ and $x_{1}(t)$ is 0.9452 and correlation coefficient of $C_{4}$ and $x_{2}(t)$ is 0.8000 by using ASTFA. Therefore, it can be concluded that ASNBD can achieve more accurate decomposition results than ASTFA and CEEMD method.

## 3. Refined Composite Multiscale Dispersion Entropy

### 3.1. Dispersion Entropy

The complexity stands for meaningful structural richness. MSE and RCMSE are the most common measures, but they are still challenges for short-term time series since the undefined values may be generated when the scale factor is large. Furthermore, their computation is not quick enough for real-time application. In [29], the refined composite multiscale dispersion entropy algorithm (RCMDE) was proposed to overcome the deficiencies. In this subsection, the dispersion entropy (DisEn) is depicted as follows.

(1) First, for a time series $x=[x_{1},x_{2},\ldots x_{N}]$ with the length N, $x_{i},i=1,2,\ldots N$ are mapped to *c* classes with integer indices from 1 to c using the normal cumulative distribution function (NCDF). Assume the NCDF maps x to $y=\left\{y_{1},y_{2},\ldots y_{N}\right\}$, that is:

(9) $$y_{i}=\frac{1}{\sigma \sqrt{2\pi }}\int_{-\infty }^{x_{i}}e^{-\frac{{\left(t-u\right)}^{2}}{2\sigma^{2}}}dt$$

where σ and u are the standard deviate and mean of time series **x**, respectively. Then, each $y_{i}$ is converted into an integer from 1 to c by using a linear algorithm, which is written as:

(10) $$z_{j}^{c}=Round(c.y_{i}+0.5)$$

where $z_{j}^{c}$ is the jth element of the classified time series. Round represents the rounding operation, which means either increasing or decreasing a number to the next digit. As a result, the time series are mapped into the class integer from 1 to c. 

(2) Time series $z_{i}^{m,c}$ are reconstructed with embedding dimension m and time delay d.

(11) $$z_{i}^{m,c}=\left\{z_{i}^{c},z_{i+d}^{c},\ldots z_{i+(m-1)d}^{c}\right\}$$

where i=1,2,…,N−(m−1)d. Then, $z_{i}^{m,c}$ is mapped into a dispersion pattern $\pi_{v_{0}v_{1}\ldots v_{m-1}}$, (v=1,2,…c, $z_{i}^{c}=v_{0}$, $z_{i+d}^{c}=v_{1}$, $z_{i+(m-1)d}^{c}=v_{m-1}$). The number of possible dispersion patterns of each reconstructed time series $z_{i}^{m,c}$ equals to $c^{m}$. And each element in $z_{i}^{m,c}$ is an integer from 1 to c.

(3) For each $c^{m}$ potential dispersion patterns, the relative frequency is computed using the equation as:

(12) $$p(\pi_{v_{0}v_{1}\ldots v_{m-1}})=\frac{number(\pi_{v_{0}v_{1}\ldots v_{m-1}})}{N-(m-1)d}$$

Note that $number(\pi_{v_{0}v_{1}\ldots v_{m-1}})$ refers to the number of dispersion patterns of $\pi_{v_{0}v_{1}\ldots v_{m-1}}$ and N−(m−1)d is the total number of embedded signals with embedding dimension m.

(4) Finally, based on the definition of Shannon’s entropy, the DisEn value is calculated as follows:

(13) $$DisEn(x,m,c,d)=-\sum_{\pi =1}^{c^{m}}p(\pi_{v_{0}\ldots v_{m-1}})\ln(p(\pi_{v_{0}\ldots v_{m-1}}))$$

From the calculation process of DisEn, it can be found that when all possible dispersion patterns have equal probability value, the irregularity degree of data is the highest, and the maximum DisEn value $\ln c^{m}$ is obtained. On the contrary, when the time series is regular or completely predictable, there is only one $\pi_{v_{0}\ldots v_{m-1}}$ different from zero and the smallest DisEn value is achieved [28].

### 3.2. Refined Composite Multiscale Dispersion Entropy

The refined composite multiscale dispersion entropy algorithm includes four main steps.

**Figure 6:**
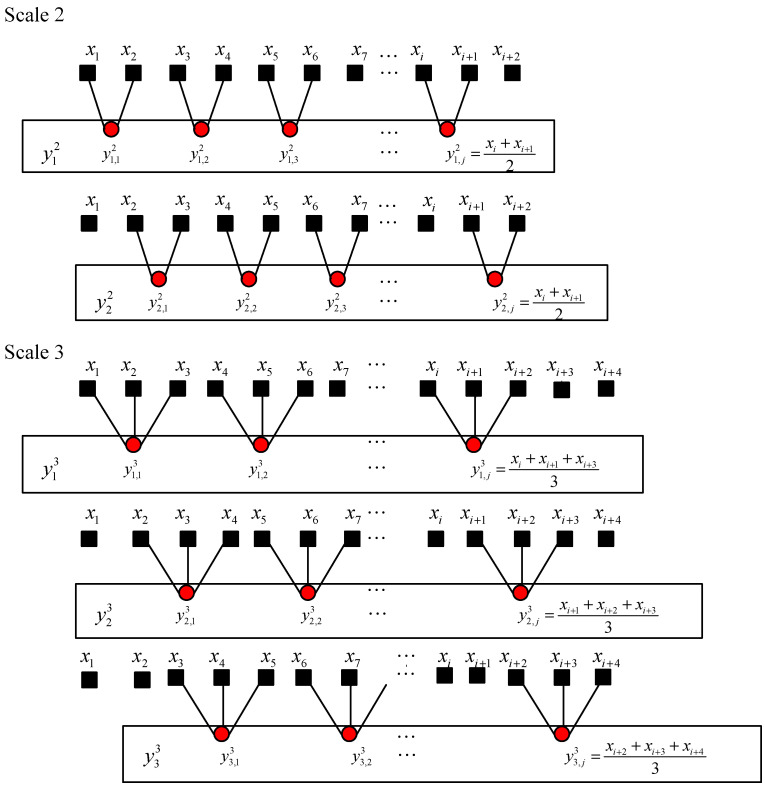
Schematic illustration of the coarse-graining procedure.

(1) To obtain coarse-grained time series at scale factor τ, the coarse-graining procedure can be demonstrated as shown in Figure 6, from which it can be seen that coarse-grained sequences are obtained from different start points. The original time series x is divided into several segments and the jth element of the kth coarse-grained time series $y_{k}^{\tau }=\left\{y_{k,1}^{\tau },y_{k,2}^{\tau },\ldots y_{k,p}^{\tau }\right\},1\leq k\leq \tau$ can be built by the following equation:

(14) $$y_{k,j}^{\tau }=\frac{1}{\tau }\sum_{i=(j-1)\tau +k}^{j\tau +k-1}x_{i},1\leq j\leq \frac{N}{\tau }$$

(2) For a scale factor τ, define the embedding dimension m and time delay d, then the relative frequency set $\left\{p_{k}^{\tau },\;,1<k\leq \tau \right\}$ of all coarse-grained time series $y_{k}^{\tau }$ are calculated as formula (12).

(3) The mean of $p_{k}^{\tau }$ is calculated by:

(15) $$\bar{p}(\pi_{v_{0}v_{1}\ldots v_{m-1}})=\frac{1}{\tau }\sum_{1}^{\tau }p_{k}^{\tau }$$

(4) Finally, RCMDE value is achieved as follow:

(16) $$RCMDE(x,m,c,d,\tau )=-\sum_{\pi =1}^{c^{m}}\bar{p}(\pi_{v_{0}v_{1}\ldots v_{m-1}}).\ln(\bar{p}(\pi_{v_{0}v_{1}\ldots v_{m-1}}))$$

### 3.3. Parameters Selection

It is an important issue to select appropriate parameters for entropy-based approach. There are four parameters, including the embedding dimension m, the number of classes c, the time delay d and the maximum scale factor τ. In general, it is recommended d=1 because when *d* > 1 some important information in terms of frequency may be discarded, which might lead to aliasing for practical work, and the number of class c must be bigger than 1, because when *c* = 1, there is only one dispersion pattern [28]. Moreover, in order to obtain reliable statistics, the number of potential dispersion patterns $c^{m}$ should be smaller than the length of the signal ($c^{m}<N$). When c is too large, a slight difference between amplitudes would change their class to obtain different dispersion entropy values, which may result in high sensitivity to noise. However, when c is too small, the amplitudes that are far from each other may be regarded as the same class and thus cause inaccurate value. When the embedding dimension m is too small, the dispersion entropy might not detect the dynamic changes. Although a bigger m can capture more information, too large m might need a longer data. In general, the length of data is between $10^{m}$ and $30^{m}$. Moreover, too large a c or m may consume more computation time. According to our research, when the parameter c is 4–10, similar results can be obtain. In addition, when the parameters m and c changed under the condition of $c^{m}<N$, the results were similar. For more information about the parameters c, m, and d, please refer to the literature [28]. For the scale factor τ, it needs to be set according to the actual situation. Simultaneously, for RCMDE, since the coarse-graining process shorten the length of a signal to Nτ, the requirement cm<Nτ must be met. 

**Figure 7:**
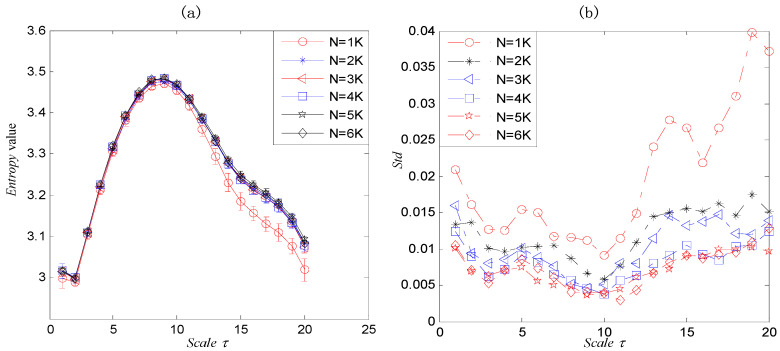
The refined composite multiscale dispersion entropy (RCMDE)value and its standard deviation of a signal with different *N*.

On the other hand, the length of datasets N will affect the estimation of the RCMDE value. Too large an N may reduce the computing efficiency. While, when N is too small, in order to satisfy the requirement that $c^{m}<N$, we have to use a smaller m or c, which likely causes the limitations described above. The capability and propriety of the RCMDE algorithm for measuring complexity was evaluated and compared with RCMSE by synthetic signals and real biomedical datasets in [29]. In order to evaluate the sensitivity of the RCMDE algorithm to the length of datasets, we employed synthetic signals for a rolling bearing with fault, which is written as shown below:

(17) $$\left\{ \begin{array}{l} x(t)=x_{1}(t)+x_{2}(t)+x_{3}(t)+x_{4}(t)\\ x_{1}(t)=\exp(-500t_{1})\sin(2\pi f_{1}t)\\ x_{2}(t)=[1+0.5\cos(40\pi t)]\sin[300\pi t+\cos(30\pi t)]\\ x_{3}(t)=2\sin(800\pi t)\\ x_{4}(t)=0.6randn(1,N) \end{array}\right.$$

where N is the length of the synthetic signal and the sample frequency is $f_{s}=12$ KHz. We employed $x_{1}(t)$ to simulate a signal of a faulty rolling bearing, in which, $f_{1}=4$ KHz, $f_{0}=30$ Hz and the periodical impulse is expressed by t1=mod(t,1fo), t is simulation time. $x_{2}(t)$ indicates the AM–FM signal, $x_{3}(t)$ is a sine signal, and $x_{4}(t)$ is white noise. According to the principle of parameter selection mentioned above, considering the calculation time and information richness, we choose the parameters as c=9, m=2, and d=1. Figure 7a,b record the statistical property of the RCMDE value changes with N, from which we can draw the following conclusion. Firstly, the entropy values have the similar trend with time scales no matter how long the dataset is. Secondly, when N is more than 2K, the obtained results are almost same. Lastly, from Figure 7b when the data length N ranges from 1K to 5K, the standard deviation (Std) decreases with N increasing, but when the data length N is more than 5000, the standard deviation goes up at some scales. Hence, based on the above analysis, we use N=2048.

## 4. Fault Diagnosis Model Proposed

**Figure 8:**
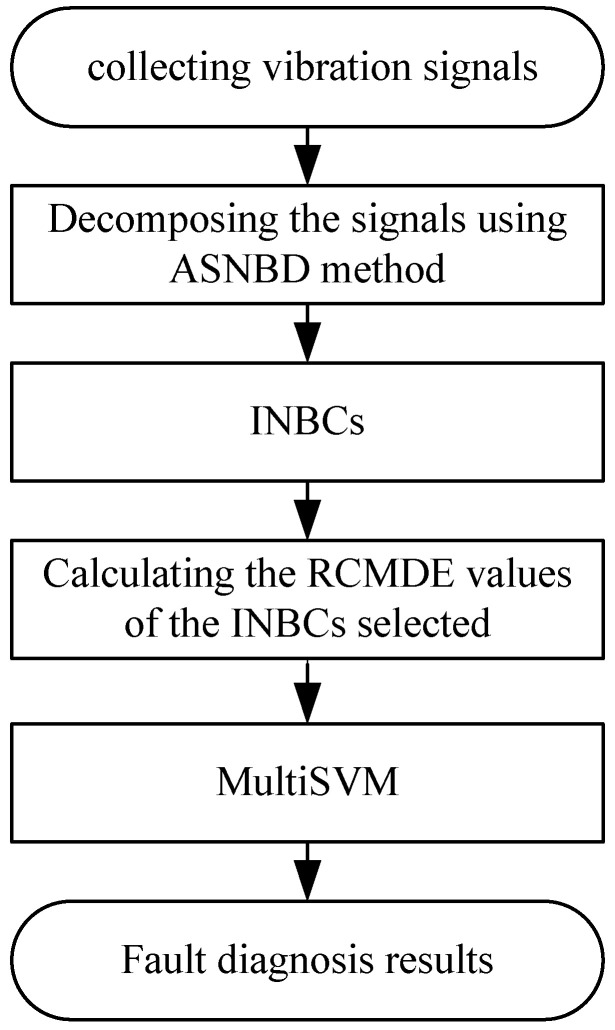
Flowchart of the proposed fault diagnosis model.

When a variety of failures occur in mechanical system, the vibration signals acquired by sensors represent the nonlinear and nonstationary characteristics and the energy distribution will change with different working states, resulting in the variety of the complexity of times series. Here, a novel-fault diagnosis-model is developed by combining the ASNBD method with the RCMDE algorithm in this paper. Firstly, a nonlinear and nonstationary vibration signal is decomposed into a series of the INBCs. Secondly, the RCMDE values from the relevant INBCs are extracted as fault features. In the end, of fault diagnosis process, basic multiSVMis employed as class discrimination technique to identify different fault type and location. The proposed fault diagnosis scheme for rolling bearing is given in Figure 8. The specific steps for the proposed scheme are given as follows.

**Step 1:** Collect $m_{i}$ vibration signals for ith classes of working states. Thus, $M=\sum_{i=1}^{k}m_{i}$ vibration signals are obtained in total for k classes.

**Step 2:** Decompose each vibration signal into several IBNCs and select the relevant INBCs, which contain rich fault information for further feature extraction.

**Step 3:** Extract the RCMDE values from selected INBCs as fault features to construct feature vectors. Suppose that n is the number of the selected INBCs, $\tau_{\max}$ is the maximum scale factor, then $n\times \tau_{\max}$ dimension of feature vectors can be achieved. Theoretically, more features are helpful to quantity fault categories from different perspectives. However, too many features may lead to huge computation cost and reduce the recognition rate. Thus, the number of INBCs used is usually set to less than four; and the maximum scale factor $\tau_{\max}$ is less than 20. Here, we set $\tau_{\max}=20$.

**Step 4:** Divide the original datasets randomly into two groups, one as the training samples, and the other for the testing samples. For an unknown test sample, failure patterns can be discriminated by the output results of the multiSVM classifier.

## 5. Application to Fault Diagnosis for Rolling Bearing

### 5.1. Datasets Collection and Signal Decomposition

**Figure 9:**
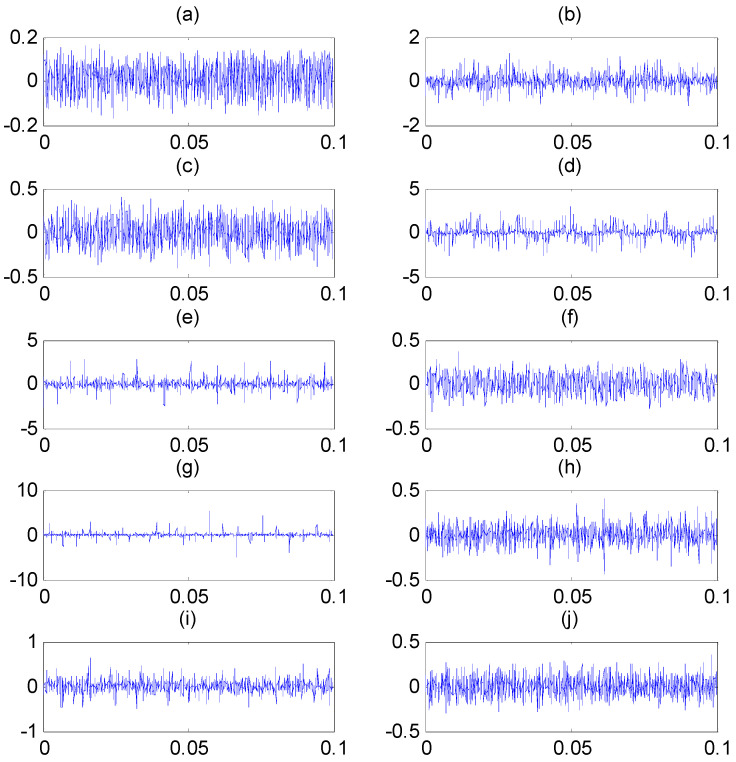
Time domain vibration signals for rolling bearing under: (**a**) normal state; (**b**) inner race fault (IRF)1; (**c**) ball fault (BF)1; (**d**) outer race fault (ORF)1; (**e**) IRF2; (**f**) BF2; (**g**) ORF2; (**h**) IRF3; (**i**) BF3; and (**j**) ORF3.

In order to verify the effectiveness, the proposed scheme is applied to the experimental datasets shared by Case Western Reverse Bearing Data Center [32]. The datasets include vibration time series collected by the accelerometer mounted on the driven-end bearings with inner race fault (IRF), ball fault (BF), outer race fault (ORF), and normal state. The driven-end bearings were charged to single-point failures with fault diameters of 0.007in to 0.021in. The sampling frequency $f_{s}$ equals to 12 KHz. The motor load is 2hp, and the shaft rotation speed is $f_{r}=1750rpm$. Ten classes of vibration signals were utilized in this paper. The datasets are divided into 55 segments as samples with the length N=2048. The more details of datasets and experimental rig are given on the Case Western Reserve University’s website. Datasets used are listed in Table 1. The time-domain waveform of vibration signals under various conditions are shown in Figure 9, from which it can be found these vibration signals are obviously nonlinear and nonstationary; and it is difficult to differ them from each other.

### 5.2. Feature Extraction by RCMDE with ASNBD

**Figure 10:**
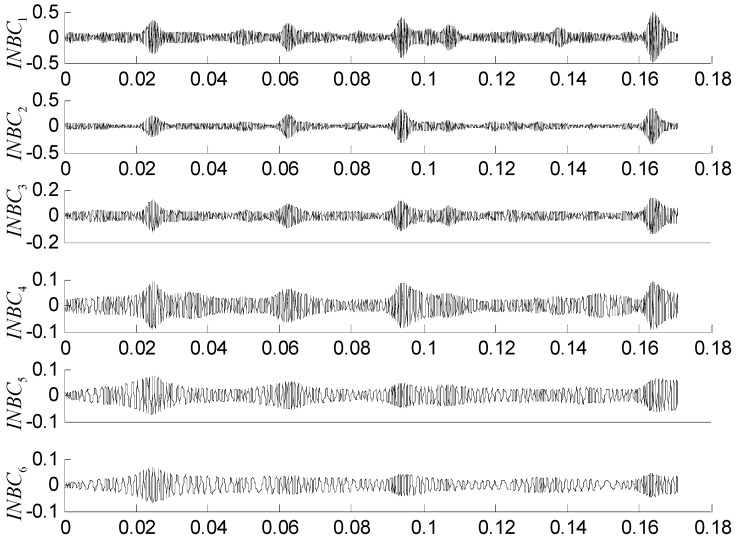
Decomposition results using the ASNBD method for vibration signal of IRF3 state.

**Figure 11:**
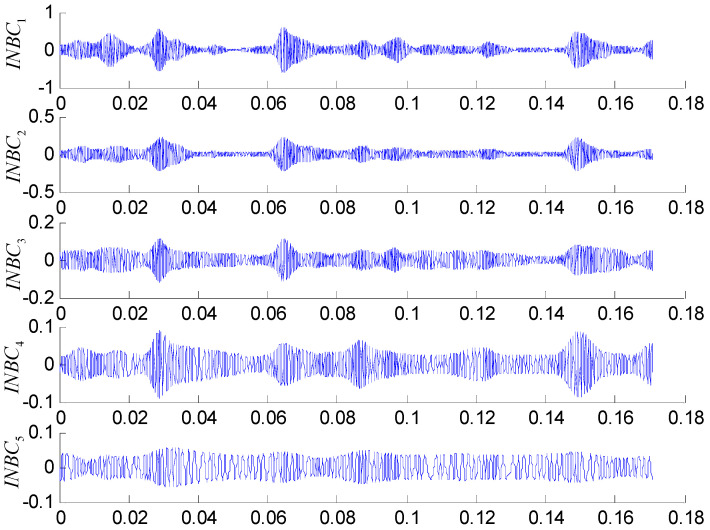
Decomposition results using the ASNBD method for a vibration signal ofBF3 state.

**Figure 12:**
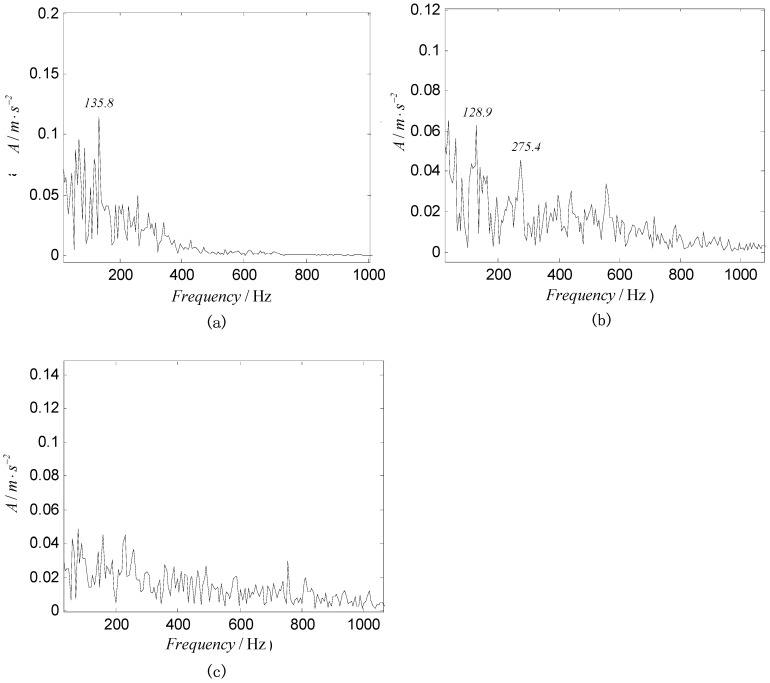
Envelop spectrums for the first component of BF signal via different methods (**a**) ASNBD, (**b**) ASTFA, and (**c**) CEEMD.

To quantify the complexity of intrinsic mode, the original vibration signal is decomposed into a number of INBCs using the ASNBD method. Simultaneously, correlation analysis is conducted between each INBC and the original signal to determine which ones are the false components. The components with small correlation coefficients are regarded as false components and removed. To the end, six–eight components are obtained as true INBCs for next analysis. Figure 10 and Figure 11, respectively, show the decomposition results for the vibration signals of IRF (noted as No.1 signal) and BF (noted as No.2 signal and please see Table 1 and Table 2). After computation, the ball-fault feature-frequency is $f_{b}=136$ Hz. Since it is most difficult to detection the ball faults, we draw the envelop spectrum for the first component of BF signal in the Figure 12, in which the ball fault frequency $f_{b}$ can be found more easily when using the ASNBD than the ASTFA and the CEEMD. This result illustrates the superiority of the ASNBD. Besides, from the abovementioned figures, it can be also concluded that fault information for rolling bearing mainly concentrates on the first several components because they present modulation and impulse characteristics with larger energy. Moreover, it is found the correlation coefficients R and kurtosis values K for the first three INBCs are bigger. Here, we list the results of correlation analysis and kurtosis values for the INBCs of No.1 signal and No.2 signal in Table 2 as an example to clarify the selecting process for better INBCs. Therefore, the first three INBCs are selected to characterize the original signal. 

**Figure 13:**
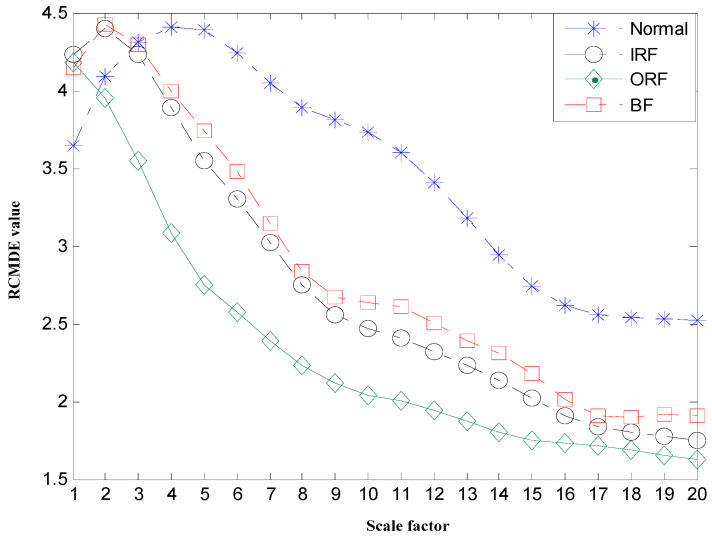
Mean value of optimal features selected of rolling bearing using RCMDE.

**Figure 14:**
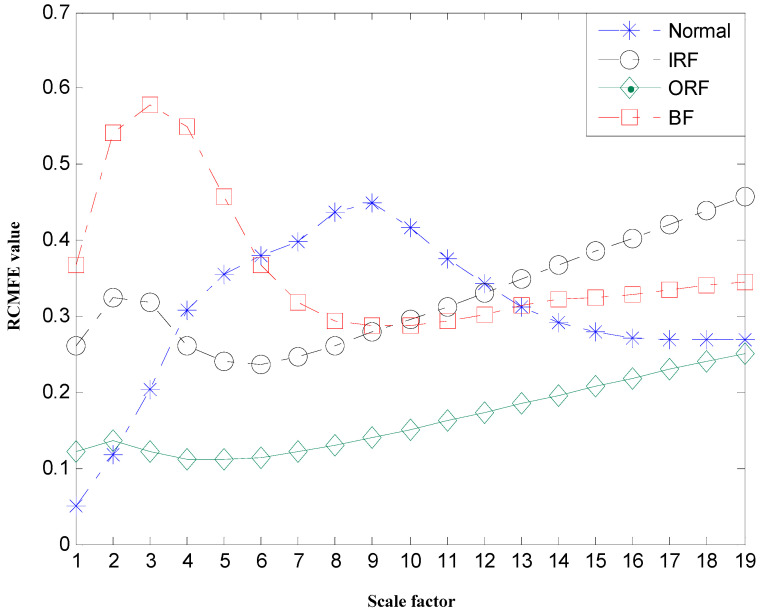
Mean value of optimal features selected of rolling bearing using RCMFE.

Subsequently, the RCMDE values for the first three INBCs are computed via the aforementioned procedure. The maximum scale is set $\tau_{\max}=20$ and the time delay d=1. Besides, considering the length of the data N=2048, and the coarse-graining process which would greatly shorten the data at large scales, the parameters c and m are set, c=6 and m=2. Accordingly, RCMDE values with 60 dimensions are achieved as fault-relevant features. For simplicity, the RCMDE values of the third INBC are given in Figure 13, from which we draw a few conclusions as follows. First of all, the RCMDE values of normal rolling bearing is much bigger in the major time scales than those under faulty states, which is consistent with the fact that the vibration signals under normal state are most complex and irregular. However, failures would change the system dynamics to become the excitation source, which will cause periodic impulse, increase the self-similarity of vibration signals, and thus drive the entropy values drop down. Secondly, the RCMDE values from vibration signals of rolling bearing under ball fault state and inner race fault state are bigger than those under outer race fault state. This phenomenon can be explained by the fact that when local failures occur in ball elements or inner race, the vibration signals would pass through a long way to the sensors which are mounted on the bearing basis, leading to more modulation components. While the outer race is fixed on the bearing basis, the pathway is shortest to the sensor and the vibration signals contain little interference, so that they show a more apparent periodical impulse and the entropy values are smaller. In addition, the RCMDE values of faulty rolling bearing monotonically decrease with the time scale increasing. This can be due to the fact than the multiscale coarse-graining procedure progressively eliminates the uncorrelated random components such that the entropy monotonically decreases with the scale factors [20]. At the same time, the RCMFE values of the third INBCs are given in Figure 14 in comparison with the RCMDE. From Figure 14, it can be found that the results fluctuate greatly. Although when the scale factor ranges from 6 to 12, the entropy values of normal rolling bearing are biggest, they have not clearly regular patterns over all scales. Most important of all, by comparing Figure 13 with Figure 14, it can be obviously shown that RCMDE leads to larger differences between various states than the RCMFE, resulting in essentially improving the fault detection rate of rolling bearing, which would be verified during the next class discrimination process.

As mentioned above, there are 55 samples for each state and there are 550 samples in total. All these samples are randomly divided into two groups, in which 100 samples (10 samples per class) are determined as training group to obtain the training matrix $T_{100\times 60}$, and 450 samples are regarded as the test group to achieve test matrix $M_{450\times 60}$. 

### 5.3. Fault Diagnosis Results and Comparison

Support Vector Machine (SVM) has excellent classification performance for small-sample recognition task. However, it is only a binary classifier and it is difficult to deal with multiple class problem. Multi-class Support Vector Machine (MultiSVM) with linear kernel function, as SVM’s extension technique, is employed in this paper. Simultaneously, to demonstrate the necessity of using the ASNBD, the RCMDE values of the raw signals are extracted and the comparison analysis is done. In addition, the RCMFEs are computed to validate the superiority of the proposed model in comparison with the RCMDEs. The results are listed in Table 3. The first row illustrates the proposed method outperforms the other approaches because it acquires the highest accuracy and the smallest standard deviation. The method shown in second row employs the RCMFE values as features instead of the RCMDE values. The third row and the fourth row use raw signals instead of INBCs to extract the RCMDE values or the RCMFE values as feature vectors. Noted that these techniques use the same class recognition method—MultiSVM to make the comparison fair. Compared the first\the third row with the second\the fourth row, it can be observed that the fault identification rate is higher and the standard deviation is lower when using the RCMDE values as input feature vectors for multiSVM classifier than using the RCMFE values. This is because RCMDE values lead to bigger difference between the bearing working states as shown in Figure 13. On the other hand, the features extracted from INBCs are more effective than those derived from raw signals from the first\second row against the third\fourth. In other words, the application of signal decomposition method is necessary to obtain more fault-sensitive features to improve the classifier’s performance.

In order to further verify the effectiveness and superiority of the ASNBD technique, we utilized the CEEMD and the ASTFA to complete signal decomposition. Similar to the proposed model, the first three intrinsic mode functions (IMFs) were used to extract the RCMDE values as features and the fault diagnosis results were listed from the fifth to the eighth rows in Table 3. In these techniques, the CEEMD or the ASTFA method was employed to preprocess the signals. No matter which signal process technique served, the entropy-based measures are effective features to fault diagnosis and can yield satisfactory results even when basic multiSVM was used. However, it is no doubt that the proposed model is best among them. At the same time, a few currently-developed techniques are listed in Table 4, from which, it can be observed that our proposed model is a promising alternative. Here, note that a satisfying classification was achieved when the moving-average based multiscale fuzzy entropy (MAMFE) combined partly ensemble local characteristic scale decomposition (PELCD)in the literature [33], but the procedure of feature extraction and selection is relatively complex and time-consuming because the MAMFE algorithm employ too many template vectors.

## 6. Conclusions 

When local faults happen, the complexity of intrinsic oscillations of a vibration signal of rolling bearing will change. In order to utilize the RCMDE value to quantify the complexity of the intrinsic oscillations at different time scales, a novel-feature extraction-technique integrated the ASNBD and the RCMDE algorithm is proposed at first in this article. Furthermore, a fault diagnosis model is built with basic multiSVM as classifier and applied to fault diagnosis for rolling bearing. We can draw the following conclusions. Firstly, since the INBCs can reveal inherent characteristics hidden in complex vibration signals, the proposed model achieves more reliable and accurate results. Secondly, when the length of dataset N ranges from 2K to 5K, the varying trend of the entropy values with time scales is almost the same, and smaller standard deviations can be obtained, which is beneficial to analyze the real-world time series in fault diagnosis practice. Thirdly, the results of the experimental analysis show the RCMDE algorithm can extract features that have bigger differences under various states than the RCMFE values, which leads to higher identification rate. Finally, the comparisons results with other existing techniques indicate that the proposed technique is feasible and effective. Simultaneously, it is worth pointing out that the proposed model can be extend to other fault diagnosis area. 

## Figures and Tables

**Table 1:** Details of datasets for rolling bearing experiment.

Class	Fault Size /in.	Fault Severity	Number of Sample	Data Length	Class Label
Inner race fault	0.007	slight	55	2048	1-IRF1
	0.014	moderate	55	2048	2-IRF2
	0.021	severe	55	2048	3-IRF3
Ball fault	0.007	slight	55	2048	4-BF1
	0.014	moderate	55	2048	5-BF2
	0.021	severe	55	2048	6-BF3
Outer race fault	0.007	slight	55	2048	7-ORF1
	0.014	moderate	55	2048	8-ORF2
	0.0021	severe	55	2048	9-ORF3
Normal			55	2048	10-Norm

**Table 2:** Correlation coefficients and kurtosis values.

	Index	$INBC_{1}$	$INBC_{2}$	$INBC_{3}$	$INBC_{4}$	$INBC_{5}$	$INBC_{6}$
No.1 signal	R	0.7946	0.4991	0.3633	0.1792	0.1078	0.0920
	K	5.5465	10.5904	3.8407	3.1361	2.8244	2.5232
No.2 signal	R	0.8452	0.3065	0.2180	0.1572	0.1270	0.1003
	K	5.6063	10.5362	3.8404	2.5615	2.0487	2.1010

Note: R indicates the correlation coefficient between the $INBC_{i}$ and the original signal and K is the kurtosis value.

**Table 3:** Fault diagnosis results.

No.	Method	Classified States	Number of Test Samples	Identification Rate (%)				Time Cost (s)
				Maximum	Minimum	Mean	Std	
1	ASNBD + RCMDE (proposed model)	10	450	100.00	100.00	100.00	0	0.20
2	ASNBD + RCMFE	10	450	100.00	97.14	99.73	0.20	0.25
3	Raw + RCMDE	10	450	100.00	96.86	99.46	0.81	0.37
4	Raw + RCMFE	10	450	96.86	81.43	89.10	3.84	0.38
5	ASTFA + RCMDE	10	450	100.00	99.78	99.89	0.16	0.13
6	ASTFA + RCMFE	10	450	100.00	96.67	98.73	1.23	0.25
7	CEEMD + RCMDE	10	450	100.00	97.78	98.90	0.64	0.28
8	CEEMD + RCMFE	10	450	99.78	95.56	98.72	1.57	0.25

**Table 4:** Comparison with a few techniques developed currently.

Method	Classified States	Number of Test Samples		Identification Rate (%)			Time Cost (s)
			Maximum	Minimum	Mean	Std	
WT + MPE [2]	4	120	/	/	94.2		/
LMD + MPE [3]	4	80	100.00	/	/	/	/
EEMD + PE [1]	11	330	/	/	97.56–99.64	0.15–0.25	/
PELCD + MAMFE [33]	10	450	100.00	100.00	100.00	0	0.28
ASNBD + RCMDE (Proposed method)	10	450	100.00	100.00	100.00	0	0.20

## Data Availability

All data included in this study are available upon request by contacting the corresponding author.

## References

[1] Zhang X.Y., Liang Y.T., Zhou J.Z., Zang Y. (2015). A novel bearing fault diagnosis model integrated permutation entropy, ensemble empirical mode decomposition and optimized SVM. Measurement.

[2] Zhao L.E., Wang L., Yan R.Q. (2015). Rolling Bearing Fault Diagnosis Based on Wavelet Packet Decomposition and Multi-Scale Permutation Entropy. Entropy.

[3] Li Y.B., Xu M.Q., Wei Y., Huang W.H. (2016). A new rolling bearing fault diagnosis method based on multiscale permutation entropy and improved support vector machine based binary tree. Measurement.

[4] Li Y.B., Xu M.Q., Wang R.X., Huang W.H. (2016). A fault diagnosis scheme for rolling bearing based on local mean decomposition and improved multiscale fuzzy entropy. J. Sound Vib..

[5] Li Y.B., Yang Y.T., Wang X.Z., Liu B.B., Liang X.H. (2018). Early fault diagnosis of rolling bearings based on hierarchical symbol dynamic entropy and binary tree support vector machine. J. Sound Vib..

[6] Tian Y., Wang Z.L., Lu C. (2019). Self-adaptive bearing fault diagnosis based on permutation entropy and manifold-based dynamic time warping. Mech. Syst. Signal Process..

[7] Zheng J.D., Pan H.Y., Cheng J.S. (2017). Rolling bearing fault detection and diagnosis based on composite multiscale fuzzy entropy and ensemble support vector machines. Mech. Syst. Signal Process..

[8] Zhang X.Y., Zhou J.Z. (2013). Multi-fault diagnosis for rolling element bearings based on ensemble empirical mode decomposition and optimized support vector machines. Mech. Syst. Signal Process..

[9] Yeh J.R., Shieh J.S., Huang N.E. (2010). Complementary ensemble empirical mode decomposition: A novel noise enhanced data analysis method. Adv. Adapt. Data Anal..

[10] Wu Z.H., Huang N.E. (2009). Ensemble empirical mode decomposition: A noise-assisted data analysis method. Adv. Adapt. Data Anal..

[11] Huang N.E., Shen Z., Long S.R. (1998). The empirical mode decomposition and the Hilbert spectrum for nonlinear and non-stationary time series analysis. Proc. R. Soc. Lond. A.

[12] Cheng J.S., Zhang K., Yang Y. (2012). An order tracking technique for the gear fault diagnosis using local mean de composition method. Mech. Mach. Theory.

[13] Peng Z.K., Wei K.X., Tian W.Y., Yang W.X. (2016). Superiorities of Variational Mode Decomposition over Empirical Mode Decomposition Particularly in Time-frequency Feature Extraction and Wind Turbine Condition Monitoring. IET Renew. Power Gener..

[14] Sahani M., Dash P.K. (2018). Variational mode decomposition and weighted online sequential extreme learning machine for power quality event patterns recognition. Neurocomputing.

[15] Yang H., Mathew J., Ma L. (2005). Fault diagnosis of rolling element bearings using basis pursuit. Mech. Syst. Signal Process..

[16] Liu X.F., Bo L., Xi X., Veidt M. (2012). Application of correlation matching for automatic bearing fault diagnosis. J. Sound Vib..

[17] Hou T.Y., Shi Z.Q. (2011). Adaptive data analysis via sparse time-frequency representation. Adv. Adapt. Data Anal..

[18] Cheng J.S., Peng Y.F., Yang Y., Wu Z.T. (2017). Adaptive sparsest narrow-band decomposition method and its applications to rolling element bearing fault diagnosis. Mech. Syst. Signal Process..

[19] Costa M., Goldberger A.L., Peng C.K. (2002). Multiscale entropy analysis of complex physiologic time series. Phys. Rev. Lett..

[20] Costa M., Goldberger A.L., Peng C.K. (2005). Multiscale entropy analysis of biological signals. Phys. Rev. E.

[21] Zhang L., Xiong G.L., Liu H.S., Guo W.Z., Zou H.J. (2010). Bearing fault diagnosis using multi-scale entropy and adaptive neuro-fuzzy inference. Expert Syst. Appl..

[22] Wu S.D., Wu C.W., Lee K.Y., Lin S.G. (2013). Modified multiscale entropy for short-term time series analysis. Phys. A Stat. Mech. Appl..

[23] Wu S.D., Wu C.W., Lin S.G., Wang C.C., Lee K.Y. (2013). Time series analysis using composite multiscale entropy. Entropy.

[24] Wu S.D., Wu C.W., Lin S.G., Lee K.Y., Peng C.K. (2014). Analysis of complex time series using refined composite multiscale entropy. Phys. Lett. A.

[25] Azami H., Fernández A., Escudero J. (2017). Refined Multiscale fuzzy entropy based on standard deviation for biomedical signal analysis. Med. Biol. Eng. Comput..

[26] Bandt C., Pompe B. (2002). Permutation Entropy: A Natural Complexity Measure for Time Series. Phys. Rev. Lett..

[27] Le D.H., Cheng J.S., Yang Y., Pham M., Thai V.T. (2017). Gear Fault Diagnosis Method Based on Local Characteristic-Scale Decomposition Multi-Scale Permutation Entropy and Radial Basis Function Network. J. Comput. Theor. Nanosci..

[28] Rostaghi M., Azami H. (2016). Dispersion entropy: A measure for time series analysis. IEEE Signal Process. Lett..

[29] Azami H., Rostaghi M., Abasolo D. (2017). Refined Composite Multiscale Dispersion Entropy and its Application to Biomedical Signals. IEEE Trans. Biomed. Eng..

[30] Chen F.F., Tang B.P., Chen R.X. (2013). A novel-fault diagnosis-model for gearbox based on wavelet support vector machine with immune genetic algorithm. Measurement.

[31] Peng S.L., Hwang W.L. (2008). Adaptive Signal Decomposition Based on Local Narrow Band Signals. IEEE Trans. Signal Process..

[32] Case Western Reserve University Bearing Data Center Website. http://csegroup.case.edu/bearingdatacenter/home.

[33] Luo S.R., Yang W.X., Luo Y.X. (2020). A Novel Fault Detection Scheme Using Improved Inherent Multiscale Fuzzy Entropy With Partly Ensemble Local Characteristic-Scale Decomposition. IEEE Access.

